# Association of Dietary Fiber Intake With Gastrointestinal Tract Cancer Among Korean Adults

**DOI:** 10.1001/jamanetworkopen.2023.4680

**Published:** 2023-03-24

**Authors:** Shinyoung Jun, Jeonghee Lee, Jeongseon Kim

**Affiliations:** 1 Department of Cancer Biomedical Science, National Cancer Center Graduate School of Cancer Science and Policy, Goyang-si, Republic of Korea; 2 Department of Cancer, AI & Digital Health, National Cancer Center Graduate School of Cancer Science and Policy, Goyang-si, Republic of Korea

## Abstract

This cohort study assesses the association between dietary fiber intake and incident gastrointestinal tract cancer among Korean adults.

## Introduction

Epidemiological studies have suggested a beneficial effect of dietary fiber on gastrointestinal tract cancers (GICs).^1,2^ In particular, the World Cancer Research Fund–American Institute for Cancer Research has recognized and promoted dietary fiber as a protective factor against colorectal cancer.^3^ However, most prospective studies on dietary fiber and GICs have been conducted in North America and Europe.^1,3^ Little is known about the association of dietary fiber with incident GIC among Koreans, although food sources of fiber and their health effects may differ across cultures and locations. The burden of GICs (ie, cancers of the esophagus, stomach, liver, pancreas, gallbladder, colon, rectum, and anus) in Korea is substantial, accounting for 36% of all cancer cases.^4^ Therefore, we assessed the association between dietary fiber intake and incident GIC among Korean adults.

## Methods

The Cancer Screenee Cohort recruited participants attending check-ups at the National Cancer Center Korea (NCC).^5^ We linked data from the Korea Central Cancer Registry and the NCC electronic medical records to identify GIC cases diagnosed from January 1, 1988, through December 31, 2019. Of 11 086 adults 20 years and older who enrolled between October 16, 2007, and December 31, 2018, we excluded 1840 who developed any cancer other than GIC, who were diagnosed with GIC within a year after enrollment, or who had implausible energy intake, or missing body mass index or physical activity information. The analysis was performed from August 1, 2022 to February 1, 2023. This cohort study was approved by the Institutional Review Board of the NCC and followed the STROBE–Nutritional Epidemiology reporting guideline. Written informed consent was obtained from all study participants.

Habitual dietary intake was measured via a validated 106-item food frequency questionnaire.^6^ Cox proportional hazards regression models were used to estimate hazard ratios (HRs) and 95% CIs for GIC or gastric cancer events comparing participants with dietary fiber intake at or above vs less than the median. Model 1 included age, sex, and total energy intake. Model 2 additionally included obesity, smoking, alcohol consumption, physical activity, comorbidity, and red and processed meat intake. A detailed description of the study design and methods can be found in the eMethods in Supplement 1. All analyses were conducted using SAS, version 9.4 (SAS Institute Inc). Two-sided *P* < .05 indicated statistical significance.

## Results

Among 9246 participants, the mean (SD) age was 52.5 (8.2) years; 5901 (63.8%) were women and 3345 (36.2%) were men (Table 1). After a median follow-up of 7.8 (IQR, 1.0-12.2) years (70 605 person-years), 163 incident GIC cases were identified. Higher intake of total dietary fiber (ie, ≥ 17.8 g/d) was associated with lower GIC incidence in model 1 (HR, 0.61 [95% CI, 0.41-0.91]) and model 2 (HR, 0.63 [95% CI, 0.42-0.93]) (Table 2). An association of total dietary fiber intake and gastric cancer was also observed (model 2 HR, 0.42 [95% CI, 0.22-0.81]). Associations of dietary fiber intake from different food sources (ie, grains, vegetables, and fruits) with GIC risks were in the protective direction but not statistically significant.

**Table 1.** Baseline Characteristics by Dietary Fiber Intake Among Participants of the Cancer Screenee Cohort^a^

Characteristic	All participants (N = 9246)	Dietary fiber intake group		*P* value^b^
		Below median (n = 4623)	At or above median (n = 4623)	
Follow-up, median (range), y	7.8 (1.0-12.2)	7.6 (1.0-12.2)	8.3 (1.0-12.2)	NA
Age, mean (SD), y	52.5 (8.2)	52.0 (8.2)	52.9 (8.1)	<.001
Sex				
Men	3345 (36.2)	1670 (36.1)	1675 (36.2)	.92
Women	5901 (63.8)	2953 (63.9)	2948 (63.8)	
BMI				
<25	6532 (70.6)	3330 (72.0)	3202 (69.3)	.004
≥25	2714 (29.4)	1293 (28.0)	1421 (30.7)	
Currently smoking	1338 (14.5)	680 (14.7)	658 (14.2)	.52
Alcohol consumption				
None	3918 (42.4)	1923 (41.6)	1995 (43.2)	.02
Moderate	4377 (47.3)	2251 (48.7)	2126 (46.0)	
Heavy	951 (10.3)	449 (9.7)	502 (10.9)	
Physical activity, MET-min/wk				
<600	2632 (28.5)	1480 (32.0)	1152 (24.9)	<.001
≥600 to <3000	4015 (43.4)	2038 (44.1)	1977 (42.8)	
≥3000	2599 (28.1)	1105 (23.9)	1494 (32.3)	
Comorbidity (any history of diabetes, heart disease, and stroke)	767 (8.3)	383 (8.3)	384 (8.3)	.97
Total energy intake, mean (SD), kcal/d	1651.2 (540.1)	1343.4 (351.9)	1979.0 (507.4)	<.001
Dietary fiber intake, mean (SD), g/d				
Total fiber	19.7 (9.4)	12.8 (3.2)	26.5 (8.5)	<.001
Fiber from grains	4.1 (1.9)	3.6 (1.4)	4.7 (2.1)	<.001
Fiber from vegetables	8.6 (5.5)	5.1 (2.2)	12.0 (5.7)	<.001
Fiber from fruits	2.7 (3.0)	1.5 (1.4)	3.9 (3.7)	<.001
Red and processed meat intake, mean (SD), g/d	50.6 (46.6)	38.8 (32.6)	62.4 (54.7)	<.001
Folate intake, mean (SD), μg/d	486.6 (237.7)	322.9 (92.9)	650.2 (225.5)	<.001

Abbreviations: BMI, body mass index (calculated as weight in kilograms divided by height in meters squared); MET, metabolic equivalent of task; NA, not applicable.

^a^ Unless otherwise indicated, data are expressed as No. (%) of participants. Percentages have been rounded and may not total 100.

^b^ Differences by dietary fiber intake were tested using the χ^2^ test for categorical variables and the Wilcoxon rank sum test for continuous variables.

**Table 2.** The Association Between Dietary Fiber Intake and Gastrointestinal Tract Cancer Among Participants of the Cancer Screenee Cohort

Dietary fiber intake^a^	Gastrointestinal tract cancer		Gastric cancer^b^	
	Model 1^c^	Model 2^d^	Model 1^c^	Model 2^d^
No. of cases/total No. of participants	163/9246	163/9246	62/9145	62/9145
Intake, HR (95% CI)				
Total fiber	0.61 (0.41-0.91)	0.63 (0.42-0.93)	0.41 (0.21-0.79)	0.42 (0.22-0.81)
Fiber from grains	0.88 (0.61-1.28)	0.87 (0.60-1.27)	0.77 (0.42-1.39)	0.76 (0.42-1.38)
Fiber from vegetables	0.76 (0.54-1.07)	0.75 (0.54-1.06)	0.76 (0.44-1.33)	0.75 (0.43-1.31)
Fiber from fruits	0.74 (0.53-1.03)	0.78 (0.55-1.08)	0.76 (0. 45-1.30)	0.82 (0.48-1.41)

Abbreviation: HR, hazard ratio.

^a^ Intake is compared between participants at or above vs below the median. The median intake values for total dietary fiber was 17.8 g/d; for dietary fiber from grains, 3.9 g/d; for dietary fiber from vegetables, 7.5 g/d; and for dietary fiber from fruits, 1.7 g/d.

^b^ Participants who developed gastrointestinal tract cancer other than gastric cancer were excluded (n = 101) when the outcome was gastric cancer, the most common type of gastrointestinal tract cancer in Korea.

^c^ Included age, sex, and total energy intake.

^d^ Included obesity (body mass index [calculated as weight in kilograms divided by height in meters squared] <25 and ≥25), smoking (smoker and nonsmoker), alcohol consumption (heavy, moderate, and none), physical activity (<600, ≥600 to <3000, and ≥3000 metabolic equivalent of task–min/wk), comorbidity (any history of diabetes, heart disease, and stroke), and red and processed meat intake (≥38.1 vs <38.1 g/d [median]) in addition to covariates in the model 1.

## Discussion

Among Korean adults, dietary fiber intake at or above the median was associated with reduced risk of GIC and gastric cancer, the most common GIC type in Korea.^4^ Our findings are generally aligned with previous findings from other geographical locations^1^ and support recommending higher dietary fiber intake for GIC prevention in Korea.

Although the Cancer Screenee Cohort provides a rare opportunity to investigate the prospective association between diet and cancer among Koreans, the relatively small sample size and short follow-up precluded analyses of specific GIC types or the application of dietary fiber cutoffs other than the median. Larger studies are needed to validate the protective effect of dietary fiber on GIC or specific GIC types.

## Notes

Supplement 1. **eMethods.** Study Population, Design, and Analyses
eReferences

Supplement 2.
Data Sharing Statement
