# Supplementary material for: Association of Dietary Fiber Intake With Gastrointestinal Tract Cancer Among Korean Adults
Source: JAMA Netw Open. 2023 Mar 24;6(3):e234680. doi: 10.1001/jamanetworkopen.2023.4680 (PMC10313144; doi:10.1001/jamanetworkopen.2023.4680)
Supplement: Supplement 1. — eMethods. Study Population, Design, and Analyses eReferences [file jamanetwopen-e234680-s001.pdf]

Supplemental Online Content

Jun S, Lee J, Kim J. Association of dietary fiber intake with gastrointestinal tract cancer among Korean adults. *JAMA Netw Open*. 2023;6(3):e234680. doi:10.1001/jamanetworkopen.2023.4680

eMethods. Study Population, Design, and Analyses

eReferences

This supplemental material has been provided by the authors to give readers additional information about their work.

© 2023 Jun S et al. *JAMA Network Open*.

eMethods. Study Population, Design, and Analyses

1. Study population

The Cancer Screenee Cohort has recruited participants from those coming to the National Cancer Center Korea for health check-ups to collect information regarding risk factors for cancer. Between October 2007 and December 2018, 11,086 participants completed both general questionnaire and food frequency questionnaire (FFQ); the response rate was 73% and the completion rate of the FFQ was 75%. We subsequently excluded 1) 1,236 participants who developed any cancer other than gastrointestinal tract cancer, 2) 256 participants who were diagnosed of gastrointestinal tract cancer within a year after enrollment, 3) 243 participants with implausible energy intake (<800 kcal or >4,000 kcal for men and <500 kcal or >3,500 kcal for women), 4) 84 participants missing body mass index, and 5) 21 participants missing physical activity information (**eFigure 1**). Therefore, the final sample included 9,243 participants.

When we compared baseline characteristics of those excluded from and included in our study, those excluded were more likely to be female, be nondrinker, and have comorbidity; were less likely to be current smoker; and had a higher age, total energy intake, dietary fiber intake from grains and fruits, and red and processed meat intake.

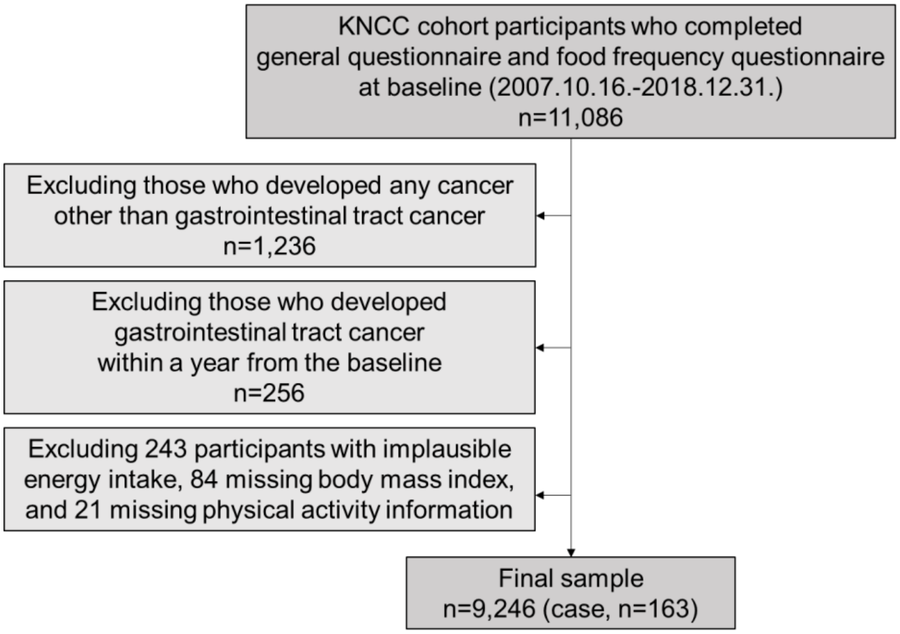

eFigure 1. Flowchart of the study sample selection

2. Case ascertainment

To identify incident gastrointestinal tract cancer cases through December 31st, 2019, we began by linking the 2019 Korea National Cancer Incidence Database. For those who could not be linked to the Korea National Cancer Incidence Database, we used Electronic Medical Records of the National Cancer Center Korea.

The gastrointestinal tract cancer was defined by International Statistical Classification of Disease and Related Health Problems, 10th revision (ICD-10) diagnostic codes: esophageal cancer (C15), gastric cancer (C16), small intestine cancer (C17), colorectal cancer (C18-C20), anal cancer (C21), liver cancer (C22), gallbladder cancer (C23-C24), and pancreatic cancer (C25). As a result, we identified 1 esophageal, 62 gastric, 1 small intestine, 49 colorectal, 27 liver, 13 gallbladder, and 10 pancreatic cancer cases.

© 2023 Jun S et al. *JAMA Network Open*.

3. Dietary assessment

Habitual dietary intake was assessed via the 106-item FFQ that was developed for Korean adults and validated against 12-day dietary records across seasons.[1] The deattenuated, age, sex, and energy intake-adjusted correlation coefficients of total energy and fiber intake were 0.43 and 0.39, respectively, between the 12-day dietary records and the second FFQ administered a year after the first FFQ.[1] Daily intakes of total energy and dietary fiber were estimated using Computer Aided Nutrition Analysis Program, version 4 (CAN-PRO 4.0, The Korean Nutrition Society, Seoul, Korea). Food groups were as defined by the Korean Nutrition Society. In brief, grains included rice, bread, and noodles; vegetables included both leafy and starchy vegetables; fruits included whole fruit and real fruit juice; and red and processed meats included beef, pork, ham, and bacon.

4. Covariate assessment

Participants completed a structured general questionnaire about demographic characteristics, lifestyle, and medical history. Smoking status was categorized as 'current smoker' and 'nonsmoker'; 5 women who were missing on smoking information were assumed as 'nonsmoker.' To determine alcohol consumption status, we calculated the number of drinks consumed per week per participant; a drink was defined as 14 g of alcohol: 350 mL of draft beer, 300 mL of rice beer, 150 mL of wine, 90 mL of 20% Soju, or 45 mL of 40% liquor.[2] Alcohol consumption was categorized as 'heavy drinker' (men with >14 and women with >7 drinks/week), 'moderate drinker' (men with >0~≤14 and women with >0~≤7 drinks/week), and 'nondrinker.'[3] 'Nondrinker' category included those who reported consuming 0 drink/week and those who were missing the number of drinks information but responded as 'never drinker or 'former drinker' to the separate question on overall alcohol consumption. Comorbidity was operationalized as any history of diabetes, heart disease, and stroke. Physical activity was assessed using the Korean version of International Physical Activity Questionnaire short form.[4] Based on the reported type, frequency, and duration of physical activity, total units of metabolic equivalent (MET-minute/week) were calculated. Participants were classified into three groups: '<600', '≥600 to <3,000', and '≥3,000' MET-minute/week, based on the recommendation by the World Health Organization[5] and the cutoff suggested by the Global Burden of Disease Study 2013[6] or the Korean version of International Physical Activity Questionnaire short form.[4]

Height and body weight were measured by trained health technicians using standard scales. Body mass index (BMI) was calculated as weight in kilograms divided by the square of height in meters; self-reported height or body weight was used when the measured height or weight was missing (n=2,442). Obesity was defined as BMI>25 kg/m^2 per WHO guidelines for the Asia-Pacific region.[7]

5. Statistical analysis

All analyses were conducted using SAS 9.4 (SAS Institute Inc., Cary, NC, USA), and two-sided *P* values below 0.05 were considered statistically significant. Baseline characteristics are presented for total participants, lower dietary fiber intake group (<median), and higher dietary fiber intake group (≥median). Differences between two dietary fiber intake groups were tested using the chi-square test for categorical variables and the Wilcoxon rank sum test for continuous variables.

For each participant, days to the event were calculated from baseline to date of gastrointestinal tract cancer diagnosis or end of follow-up (December 31st, 2019), whichever was earlier. Cox proportional hazard regression models were used to estimate HRs and 95% confidence intervals (CIs) for events (gastrointestinal tract cancer or gastric cancer, the most common gastrointestinal tract cancer type) comparing participants with dietary fiber intake ≥ and < the median; the lower intake group served as the reference group. The proportional hazards assumption was tested by including an interaction term between the covariate and the time in the model, and no serious violations of the assumptions were observed ($P > 0.05$).

© 2023 Jun S et al. *JAMA Network Open*.

Multivariate models included a priori selected covariates. The minimal model (model 1) only included age, sex, and total energy intake. The full model (model 2) included the following covariates in addition to those in the minimal model: obesity (body mass index <25 and ≥25 kg/m2), smoking (smoker and nonsmoker), alcohol consumption (heavy, moderate, and nondrinker), physical activity (<600, ≥600~<3,000, and ≥3,000 MET-minutes/week), comorbidity (any history of diabetes, heart disease, and stroke), and red and processed meat intake (≥ and < the median). Folate intake was very strongly correlated with dietary fiber intake (r=0.95) and, thus, could not be added. Sensitivity analyses were conducted by further adjusting for education level or postmenopausal status (only among women); neither variable meaningfully altered the results, so data were not presented.

eReferences

1. Ahn Y, Kwon E, Shim JE, et al. Validation and reproducibility of food frequency questionnaire for Korean genome epidemiologic study. *Eur J Clin Nutr*. 2007;61(12):1435-41. doi:10.1038/sj.ejcn.1602657
2. Lee S, Kim JS, Jung JG, Oh MK, Chung TH, Kim J. Korean Alcohol Guidelines for Moderate Drinking Based on Facial Flushing. *Korean J Fam Med*. 2019;40(4):204-211. doi:10.4082/kjfm.19.0059
3. Byrd DA, Judd SE, Flanders WD, Hartman TJ, Fedirko V, Bostick RM. Development and Validation of Novel Dietary and Lifestyle Inflammation Scores. *J Nutr*. 2019;149(12):2206-2218. doi:10.1093/jn/nxz165
4. Oh JY, Yang YJ, Kim BS, Kang JH. Validity and reliability of Korean version of International Physical Activity Questionnaire (IPAQ) short form. *Korean J Fam Med*. 2007;28(7):532-541.
5. World Health Organization. *Global Physical Activity Questionnaire (GPAQ) Analysis Guide*. Accessed September 10, 2022. https://cdn.who.int/media/docs/default-source/ncds/ncd-surveillance/gpaq-analysis-guide.pdf?sfvrsn=1e83d571_2
6. Kyu HH, Bachman VF, Alexander LT, et al. Physical activity and risk of breast cancer, colon cancer, diabetes, ischemic heart disease, and ischemic stroke events: systematic review and dose-response meta-analysis for the Global Burden of Disease Study 2013. *BMJ*. 2016;354:i3857. doi:10.1136/bmj.i3857
7. World Health Organization. *The Asia-Pacific perspective: redefining obesity and its treatment*. World Health Organization; 2000.

© 2023 Jun S et al. *JAMA Network Open*.
