# Supplementary material for: Association of Dietary Fiber Intake With Gastrointestinal Tract Cancer Among Korean Adults
Source: JAMA Netw Open. 2023 Mar 24;6(3):e234680. doi: 10.1001/jamanetworkopen.2023.4680 (PMC10313144; doi:10.1001/jamanetworkopen.2023.4680)
Supplement: Supplement 2. — Data Sharing Statement [file jamanetwopen-e234680-s002.pdf]

Data Sharing Statement

Jun. Association of Dietary Fiber Intake With Gastrointestinal Tract Cancer Among Korean Adults. *JAMA Netw Open*. Published March 24, 2023.
doi:10.1001/jamanetworkopen.2023.4680

Data
Data available: Yes
Data types: Data dictionary
How to access data: jskim@ncc.re.kr
When available: With publication

Supporting Documents
Document types: None

Additional Information
Who can access the data: anyone requesting the data dictionary
Types of analyses: for research purpose
Mechanisms of data availability: without investigator support
Any additional restrictions: To protect participant privacy, we cannot publicly post individual-level data. Qualified researchers with a valuable research question and relevant approvals including ethical approval can request access to the de-identified data from the corresponding author.
